# Editorial for “Concordance Between MR Safety Guidance Documents on Conditions for a Safe MR Environment”

**DOI:** 10.1002/jmri.70508

**Published:** 2026-08-12

**Authors:** Love Engström Nordin, Karin Åberg, Peter Lundberg

**Affiliations:** ^1^ Department of Neurobiology, Care Sciences and Society (NVS) Karolinska University Hospital, Karolinska Institutet Stockholm Sweden; ^2^ Department of Diagnostic Medical Physics Karolinska University Hospital, Karolinska Institutet Stockholm Sweden; ^3^ Center for Experimental and Biomedical Imaging in Örebro (CEBIO), Faculty of Medicine and Health, Örebro University Örebro Sweden; ^4^ Department of Radiology and Medical Physics Örebro University Hospital Örebro Sweden; ^5^ Clinical Department of Radiology in Linköping Region Östergötland Linköping Sweden; ^6^ Center for Medical Image Science and Visualization Linköping University Linköping Sweden; ^7^ Department of Health, Medicine, and Caring Sciences Linköping University Linköping Sweden; ^8^ Clinical Department of Medical Radiation Physics Region Östergötland Linköping Sweden

The systematic analysis in a recent review study about identifying consensus conditions for a safe MR environment [1], provides a timely and much needed overview of how national MR safety guidance documents address the physical MR environment. By comparing six nationally recognized documents across facility design, MR system features, and peripheral equipment, the authors demonstrate encouraging convergence: consensus was achieved on most conditions evaluated. This is reassuring, yet the gaps identified point toward several challenges that the MR safety community still needs to resolve.

The authors note that facility design acts as an engineering control for MR safety, whereas lacking design forces a reliance on weaker administrative substitutes [2]. This framing highlights that safety is an environmental feature, not just a matter of staff compliance. However, even the best facility design underperforms without structural support. A robust management system for MR safety is therefore essential to actively guide practice—regardless of whether the facility is public, private, clinical, or research‐focused.

The study also highlights the persistent lack of robust incident data. Severe underreporting of MR incidents has been documented in multiple countries, including Sweden and Denmark [3, 4, 5]. Without systematic, mandatory national reporting infrastructure, the evidence base for updating guidance remains very thin and reactive. Countries with mature and modern patient safety frameworks and effective national incident reporting systems should be better positioned to generate the feedback loops needed to refine guidance over time, though this remains to be studied in the MR safety context. National MR safety guidance documents should explicitly endorse participation in national mandatory adverse event reporting systems, which is a necessity for evidence‐based guidance.

Another systemic gap rarely addressed in national guidance is the heterogeneity of implant safety information management. Decisions about scanning patients with implants are often complex. Locally maintained lists, informal departmental protocols, and reliance on individual clinician knowledge are fragile and often difficult to standardize as well as to maintain. National guidance should address minimum standards for how implant‐related decisions are documented, communicated, and reviewed across departments.

The article focuses primarily on what guidance says about the existing MR environment. Equally important is guidance for new installations. Architects, hospital planners, and procurement staff rarely have sufficient clinical MR safety expertise. Explicit planning requirements, minimum room specifications (including size), and early involvement of MR safety expertise in construction projects could substantially reduce the structural deficits that clinical teams later need to compensate for administratively [6, 7].

A structural ambiguity present in many guidance documents concerns the distribution of responsibility between individual professionals and the organization. Who is accountable when a consensus condition is not met, the MR technologist, the MR safety officer/expert, the department head, the hospital administration? Clarity on this distinction matters. Professional responsibility, the ethical and legal obligations of the individual employee, must be distinguished from organizational accountability, which encompasses the management system within which staff members operate. Healthcare regulations in several countries now require formal quality management systems for clinical activities and MR safety programs should similarly be framed within these structures [8].

Effective MR safety practice relies on functional collaboration among various professionals, such as radiologists, radiographers, and physicists. While the article focuses on physical environment conditions, the broader challenge is how national guidance translates into multiprofessional responsibility. There is practical value in operational guidance that clearly delineates roles without creating contradictory hierarchies. Straightforward, role‐specific guidance that is concise and actionable may serve clinical teams better than comprehensive reference documents that are difficult to consult under operational pressure.

The authors note a tendency for more recent documents to show higher alignment with consensus conditions. This is encouraging, but the pace of change in MR technology, clinical applications, and patient populations demands that guidance be treated as a living document rather than a periodic publication. Mechanisms for rapid, evidence‐triggered updates, informed by incident data, implant registry findings, and emerging research, should be built into the governance structure of national guidance from the start. The recent update of the Swedish national recommendations exemplifies this principle in practice [9, 10].

The review study about identifying consensus conditions for a safe MR environment [1] provides an important baseline. The MR safety community now has a clearer picture of where guidance converges and where it diverges. The next step is to move beyond cataloguing conditions toward building the institutional, professional, and regulatory infrastructure that makes those conditions reliably achievable. MR accidents are expensive; let us work together to eliminate them.
